# Structural and biological investigation of chitosan/hyaluronic acid with silanized-hydroxypropyl methylcellulose as an injectable reinforced interpenetrating network hydrogel for cartilage tissue engineering

**DOI:** 10.1080/10717544.2021.1895906

**Published:** 2021-03-19

**Authors:** Mu Hu, Jielai Yang, Jihai Xu

**Affiliations:** a Department of Orthopedics, Ruijin Hospital North, School of Medicine, Shanghai Jiaotong University, Shanghai, China; b Department of Orthopedics, Ruijin Hospital, Shanghai Jiao Tong University School of Medicine, Shanghai, China; c Department of Hand Surgery, Ningbo No. 6 Hospital, Jiangdong, Ningbo, China

**Keywords:** CS/HA/Si-HPMC hydrogel, cartilage repair, chondrocytes, tissue regeneration, biomedical applications

## Abstract

Cartilage damage continues to pose a threat to humans, but no treatment is currently available to fully restore cartilage function. In this study, a new class of composite hydrogels derived from water-soluble chitosan (CS)/hyaluronic acid (HA) and silanized-hydroxypropyl methylcellulose (Si-HPMC) (CS/HA/Si-HPMC) has been synthesized and tested as injectable hydrogels for cartilage tissue engineering when combined without the addition of a chemical crosslinking agent. Mechanical studies of CS/HA and CS/HA/Si-HPMC hydrogels showed that as Si-HPMC content increased, swelling rate and rheological properties were higher, compressive strength decreased and degradation was faster. Our results demonstrate that the CS and HA-based hydrogel scaffolds, especially the ones with 3.0% (w/v) Si-HPMC and 2.5/4.0% (w/v) CS/HA, have suitable physical performance and bioactive properties, thus provide a potential opportunity to be used for cartilage tissue engineering. *In vitro* studies of CS/HA and CS/HA/Si-HPMC hydrogels encapsulated in chondrocytes have shown that the proper amount of Si-HPMC increases the proliferation and deposition of the cartilage extracellular matrix. The regeneration rate of the CS/HA/Si-HPMC (3%) hydrogel reached about 79.5% at 21 days for long retention periods, indicating relatively good *in vivo* bone regeneration. These CS/HA/Si-HPMC hydrogels are promising candidates for tissue compatibility injectable scaffolds. The data provide proof of the principle that the resulting hydrogel has an excellent ability to repair joint cartilage using a tissue-engineered approach.

**RESEARCH HIGHLIGHTS**
- An injectable hydrogel based on CS/HA/Si-HPMC composites was developed.
- The CS/HA/Si-HPMC hydrogel displays the tunable rheological with mechanical properties.
- The CS/HA/Si-HPMC hydrogel is highly porous with high swelling and degradation ratio.
- Increasing concentration of Si-HPMC promote an organized network in CS/HA/Si-HPMC hydrogels.
- Injectable CS/HA/Si-HPMC hydrogels have a high potential for cartilage tissue engineering.

## Graphical Abstract

**Figure:**
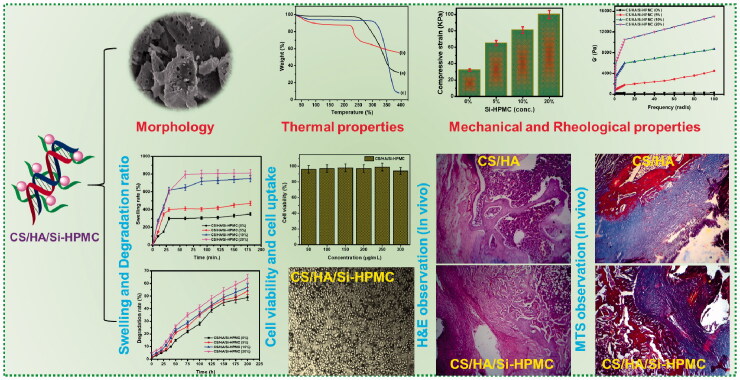

## Introduction

Cartilage is a highly specialized connective tissue that has restricted capacity for self-healing because of its compositional and structural nature, a condition that contributes to regeneration after injury (Zhang et al., 2009). Unfortunately, due to vascularity and a low supply of repair cells in this tissue, human cartilage has a restricted capacity to repair itself. To treat cartilage defects, numerous surgical techniques have been attempted, but they have not yet proved to be successful. There is also an intense clinical need for new methods to induce the regeneration of cartilage. By using biocompatible hydrogels in combination with cells and bioactive molecules for cartilage repair, tissue engineering provides an alternative approach in this regard (Liu et al., 2017). The use of scaffolds, which can promote the growth, proliferation, and differentiation of integrated chondrocytes and/or progenitor cells, usually involves this approach (Bružauskaitė et al., 2016). They mimic hydrated natural cartilage tissue and are considered ideal scaffolds for cartilage tissue engineering, as hydrogels are elastic networks with high water content (Kim et al., 2011). For several biomedical applications, various hydrogels and microspheres have been used as injectable scaffolds (Yan et al., 2016; Aswathy et al., 2020). As delivery devices, cell carriers, and tissue-engineered scaffolds, injectable and biodegradable hydrogels can be used, enabling simple and homogeneous delivery of drugs or cells of any defect size or form (Tan & Marra, 2010; Li et al., 2019; Mantha et al., 2019).

Hydrogels, considering their injectability and their adjustable mechanical and biochemical properties, are especially interesting scaffolds for the repair of cartilage (Hunt et al., 2014; Chuah et al., 2017; Wu et al., 2020). The role of chitosan (CS) has been widely documented for its biocompatible properties in tissue engineering and drug delivery (Ahsan et al., 2018). Several studies have shed light on the applications of CS. Hyaluronic acid (HA) is a linear polysaccharide that is present in the articular cartilage and is involved in controlling cell functions, including fostering the phenotype of chondrogen and generating and maintaining the components of the matrix (Kim et al., 2011; Mahapatra et al., 2016; Liu et al., 2017; Zhu, Wang, et al., 2017). HA shows promise in applicable biomedical hydrogel systems because of its good biocompatibility, biodegradation, and excellent gelling characteristics (Tan et al., 2009; Burdick & Prestwich, 2011; Khunmanee et al., 2017). Since the removal of injected HA is rapid, the HA is generally cross-linked to last longer. However, the HA hydrogel has drawbacks such as a fast degradation rate, a rather complicated crosslinking process, and poor mechanical and physical properties. HA is also involved in tissue repair and wound healing processes due to its antioxidant properties (for example, its ability to remove free radicals). Due to the many advantages of HA, many studies have explored different techniques for incorporating HA into cartilaginous tissue-engineered scaffolds, including entrapment in collagen or alginate scaffolds, adhesion to skin surfaces scaffolding, incorporation into fibers, or chemical functionalization to allow crosslinking (Tan et al., 2009; Burdick & Prestwich, 2011; Mahapatra et al., 2016; Khunmanee et al., 2017; Zhu, Wang, et al., 2017).

In recent decades, cellulose and its derivatives have been widely used, primarily because of their possible applications in various fields and properties, such as biodegradability, water solubility (derivatives), film-forming capabilities, etc (Sannino et al., 2009). The most important hydrophilic support material used in the preparation of an oral managed drug delivery system is hydroxypropyl methylcellulose (HPMC), such as a natural, water-soluble, non-toxic polymer with different desirable properties (Ghosal et al., 2011; Sahoo et al., 2015). The high swellability, which has a major effect on the release kinetics of the integrated drug, is one of the main features. The physicochemical properties of this polymer are highly affected by (i) the methoxy group content; (ii) the hydroxypropyl group content; and (iii) the weight of the molecules. To achieve this aim, we are developing an injectable hydrogel based on a silanized cellulose derivative, silated-hydroxypropyl methylcellulose (Si-HPMC), for cartilage tissue engineering (Vinatier et al., 2005; Martin et al., 2008; Rusu et al., 2019). Si-HPMC with suspended alkoxysilane or siloxide groups enables the sol-gel path to be reversible and pH-dependent poly-condensed, enabling the injected hydrogel to adjust and remain in the damaged region (Joshi, 2011). As per previous reports, Si-HPMC does not necessitate toxic components and catalysts to harden *in situ*. Particularly, these type of new generation gels have self-hardening ability without loss of their basic properties from the first-generation gel, which significantly depends on silanol condensation on the HPMC gel. Generally, the process of silanol has superior advantages in conventional chemistry and provides greater biocompatible biomaterials, and also influences the linkages on surface modifications. In addition, some research reports have elaborated that the presence of silanol has an inducing bone-like apatite formation and greatly favorable for the bone repair mechanisms *in vivo*, which is confirmed that silicon could have significantly played in the chelation between calcium and silanol (bone calcification) (Viguier et al., 2016; Buchtová et al., 2018). In bone tissue engineering, the interaction of CS/HA and Si-HPMC is hydrogen-bonded and emphasized.

**Scheme 1.**
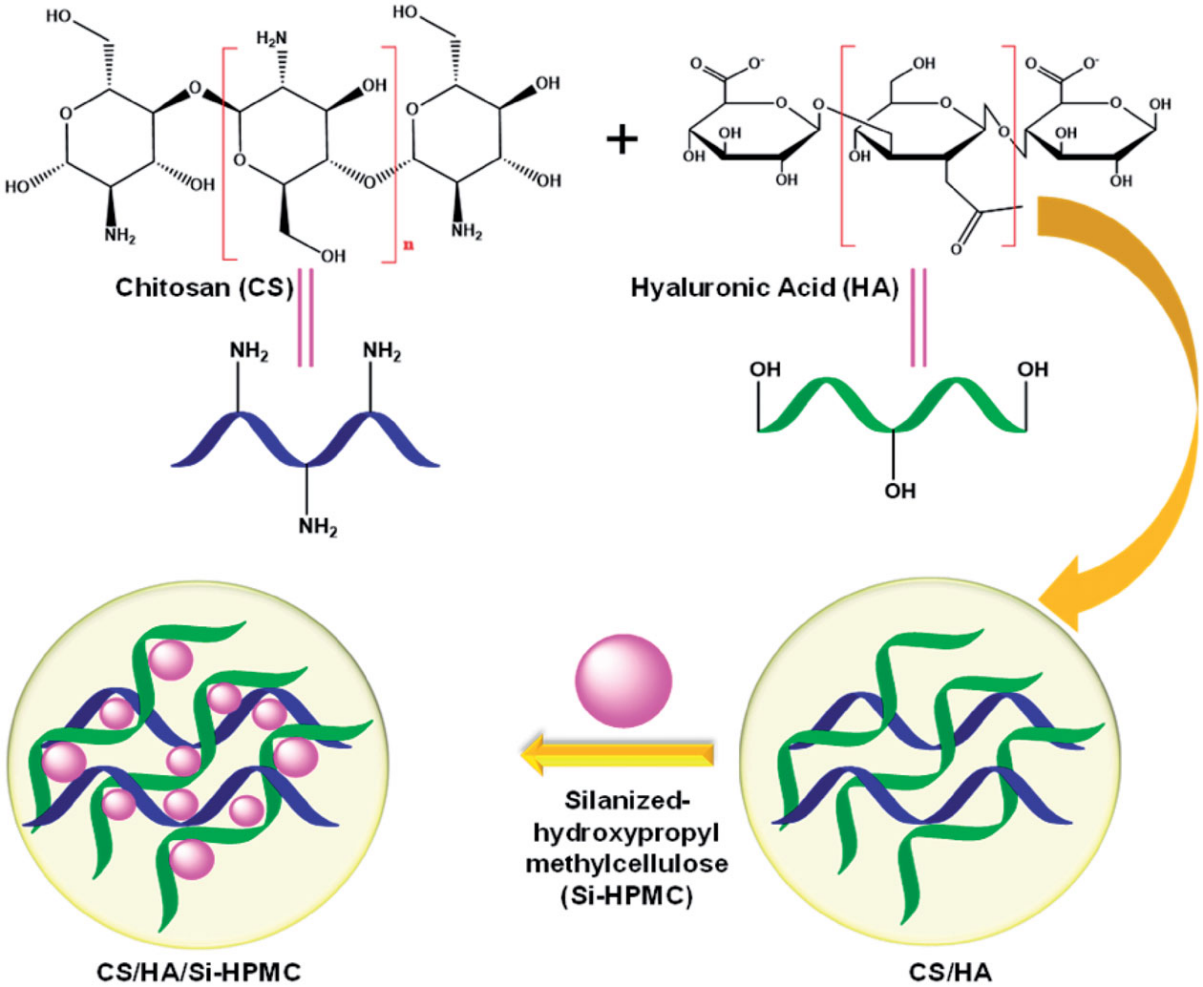
Schematic illustration of the preparation procedures of Si-HPMC incorporated CS/HA injectable hydrogel.

The incorporation of various concentrations of Si-HPMC in the CS/HA hydrogel to obtain a variable composite scaffold of CS/HA/Si-HPMC by the lyophilization method is recorded for the first time in the present research (Scheme 1). To our knowledge, this has not been reported to date. We hypothesized that the incorporation of Si-HPMC would improve the performance of CS/HA hydrogels in tissue engineering of cartilage. The manufactured CS/HA and CS/HA/Si-HPMC hydrogels were characterized by performing morphological analyzes and FTIR, XRD, and TGA. The rheological properties, compressive strength, swelling rate, and degradation rate were studied to confirm the mechanical properties of CS/HA and CS/HA/Si-HPMC hydrogels. Cell compatibility, accompanied by cell viability and absorption, was assessed by the MTT assay. Furthermore, we investigated the ability of the CS/HA/Si-HPMC hydrogel to *in vivo* form cartilage tissue regeneration in nude mice and a histological staining study was performed.

## Experimental details

### Materials

The CS (weight: 188 kDa, DD: 80%) was obtained from shrimps. HA sodium, acetic acid, sodium hydroxide, sodium ascorbate were purchased from Sigma-Aldrich. 3-Glycidoxypropyl trimethoxysilane (Si-HPMC; molar mass: 4.2 × 108 g/mol; the degree of substitution: 0.7) was purchased from Sigma-Aldrich. All other reagents are commercially available and used as received.

### Preparation CS/HA hydrogel

A 0.5 g/L CS solution was prepared for the hydrogel preparation by dissolving the CS powder in 2% by volume acetic acid and stirring until the powder was completely dissolved. The beaker was covered and sealed before mixing for 1 h to achieve complete dissolution at room temperature. A stock gel of 2.5% CS in 0.1 M acetic acid was prepared and by adding 1 M NaOH dropwise, the pH was changed from 6.0 to 8.0. In the CS solution, sodium ascorbate (1.64 mg, 0.01 mmol) was applied, then the HA (5%) mixture and the CS sodium ascorbate solution were vigorously mixed with a vortex mixer. The formulation was left overnight at 4 °C resulting in complete hydration of the polymer (Zhang et al., 2016). The resulting hydrogel was mixed at 300 rpm with a spiral stirrer for 10 min. The product was dialyzed (3500 MWCO) and lyophilized for 2 days against deionized water.

### Synthesis of porous CS/HA/Si-HPMC scaffolds

As mentioned above, the synthesis of Si-HPMC was carried out in a heterogeneous medium by inoculating 11% of 3-glycidoxypropyl trimethoxysilane on E4 M^®^. The Si-HPMC powder (3% w/v) was dissolved for 48 h at 0.2 M NaOH with regular stirring. The solution was then steam sterilized (150 °C, 30 min). The solution was finally mixed with a 0.5 volume buffer of 0.26 M HEPES to allow the formation of a crosslinked hydrogel (Liu et al., 2014). As specified by the following method, the CS/HA/Si-HPMC scaffold was prepared. In a typical procedure (Boyer et al., 2018), the CS powders were first mixed with the HA powders. Then the mixture was gradually added to 0.1 M acetic acid at 50 °C and stirred at room temperature until the transparent solution was obtained. 2 M NaOH was used to adjust the pH of the solutions to 7.4. Then, to prepare sample solutions, different amounts of Si-HPMC were applied dropwise to the solutions with continuous stirring. Stir the mixture for 8 h or until a thick paste is fully blended and formed. Sonicate the mixture before applying the suspension to the pre-designed molds to allow the distribution of Si-HPMC uniformly. Then, to freeze the solvent, the solidified mixture was held overnight in a −30 °C freezer. Finally, in a freeze dryer, the sample was lyophilized until it was cleaned, and a scaffold was reached. The scaffold was soaked in 0.2 mol/L NaOH solution for several hours to extract the acetic acid residue, then washed in deionized water and dried at 60 °C in a vacuum oven.

### Morphological measurement

The surface morphology of the CS/HA, Si-HPMC, and CS/HA/Si-HPMC composite scaffolds was examined by field emission scanning electron microscopy (FESEM) (FEI Co., Europe) operating at a voltage of 10 kV acceleration. The hydrogels were pre-frozen at −80 °C, then lyophilized for 24 h, cut with a knife, glued to the conductive plate, and spray gold plated.

### FTIR analysis

Using an FTIR spectrometer (IRAffinity-1, Shimadzu, Japan), the functional groups of the composite hydrogels were characterized. To create a homogeneous fine powder, the dried samples were ground with KBr and then pressed into a pellet. All samples were analyzed as a powder, thoroughly blended with KBr powder, compressed into a 1 mm clear pellet, and analyzed at room temperature in an attenuated total reflection mode with a resolution of 4 cm^−1^ within the 4000–400 cm^−1^ range.

### X-ray diffraction (XRD)

XRD patterns of composite hydrogel arrays in the 2*θ* scanned angular range from 10 to 80° were collected using PANalytical X’Pert3 powder (PANalytical, Netherlands) fitted with a CuKa radiation source, actuated (40 kV and 40 mA) at a scanning speed of 6°/min. The sample was put in liquid nitrogen for 4 h before being subjected to XRD analysis and ground into a fine powder with a mortar and pestle.

### Thermogravimetric analysis

The thermal stability and thermal decomposition properties of the hydrogels were evaluated by thermal gravimetric analysis (TGA; TA-Q500). The hydrogels were instantly frozen at −80 °C, followed by lyophilization, and then ground into powder. The CS/HA and CS/HA/Si-HPMC hydrogel were heated in an aluminum pan at a heating rate of 10 °C/min from 50 to 450 °C under a nitrogen atmosphere.

### Mechanical properties

A tensile tester (INSTRON 3365, Norwood, MA, USA) was used to assess the tensile efficiency of the CS/HA and CS/HA/Si-HPMC composite hydrogels. A cylindrical mold with a diameter of 13 mm was used to create the hydrogel. The developed hydrogel was cut to a thickness of 1 cm and put in a Bose Electro Force (high-precision biomaterial testing machine). By dividing the area under the stress-strain curves, durability was measured. For each sample, the mechanical properties are based on the average value of 5 samples.

### Rheological analysis

All rheological experiments were performed with a rheometer (DHR-1, TA Instruments, USA) using parallel plates (plates diameter: 40 mm, plates distance: 500 nm) at 37 °C in oscillating mode. At a frequency of 1 Hz and an elongation of 1%, the calculation is continued until the storage modulus (G′) reaches a plateau value. In a frequency sweep, samples were kept for 60 min between parallel plates, with a gap of 1 mm. At a constant voltage of 1%, the frequency sweep was carried out in the range of 0.1–100 Hz. The amplitude sweep at 1 Hz was between 0.1% and 500% elongation.

### Compression tests

The hydrogels were shaped on 24-well plates and were permitted for 24 h to crosslink. Universal Testing Systems (Instron 5969, Instron Instruments, USA) were used to measure the compressive modulus of hydrogels of known dimensions (height and diameter) with a 1 g cylinder load moving at 0.5 mm/s to the force (Pa) to expand according to the stretch. Since the initial surface of the cylindrical hydrogel sample was not completely smooth, between 5% and 20% elongation, the compressive modulus was measured as the curve slope.

### Swelling ratio of the CS/HA/Si-HPMC composite hydrogels

Strong swelling is one of the most beneficial properties of hydrogels. The rate of swelling of the composite CS/HA and CS/HA/Si-HPMC hydrogels was measured by the ambient temperature weighing method. The swelling behavior of the lyophilized hydrogels was observed for 3 h. In detail, pre-weighed dry CS/HA and CS/HA/Si-HPMC composite hydrogels were submerged in an aqueous solution until swelling to equilibrium. Swollen hydrogels were removed and immediately weighted with a microbalance after filter paper absorbed excess water that remained on the surfaces.

(1) $$\text{Swelling ratio}=W_{t}-W_{0}/W_{0}\;\times \;100\%$$

where *W_t_* is the swollen state weight of the sample at equilibrium and *W*_0_ is the dry state weight of the sample. Three samples were tested using Equation (1), and the data were reported as statistical mean and standard deviation.

### *In vitro* degradation of the CS/HA/Si-HPMC composite hydrogels

To assess weight loss due to degradation, *in vitro* degradation studies of the composite CS/HA and CS/HA/Si-HPMC hydrogels were conducted. First, 1.0 mL of each sample was put into a tube and incubated for 3 h at 37 °C to complete the gelation. Then 6.0 mL of PBS (pH∼7.4) containing the enzyme alpha-amylase was applied to the tube. Under shaking conditions (37 °C), the sample solution was maintained for another period (0–200 h). 1 mL of sample solution was taken from the tube at each interval and balanced with a fresh medium immediately. A ninhydrin assay has determined the existence of free amino acids. Finally, the solutions were measured by a Lambda 35 UV-Vis spectrophotometer (Perkin-Elmer, USA) for their absorbance at 570 nm. Afterward, the samples were lyophilized and weighed (*W_t_*). The percent weight loss (*W*%) was used to calculate the hydrogel degradation under the following Equation (2):

(2) $$\text{Degradation ratio }(W\%)=W_{0}\;-\;W_{t}/W_{0}\;\times \;100$$

All the experiments were performed in triplicate.

### Cytotoxicity of the hydrogel formulations

Analysis of cytotoxicity activity with 3-(4,5-dimethyl-2-thiazolyl)-2,5-diphenyl-2H-tetrazolium bromide (MTT, Sigma-Aldrich). L929 fibroblast cells (Shanghai Bioleaf Biotech Co., Ltd., China) were adopted and cultured with 10% (v/v) fetal bovine serum, 100 U/mL penicillin, and 100 μg/mL streptomycin (Gibco, USA) at 37 °C and 5% CO_2_ in Dulbecco’s Modified Eagle’s Medium (DMEM, Gibco, USA). In a 96 well plate containing DMEM under controlled environmental conditions (temperature 37 °C and 5% CO_2_ concentration), cells (1 × 10^6^ cells/mL) were grown individually for 24 h. Then, the supernatant was replaced by 100 μL of the CS, HA, Si-HPMC, CS/HA, and CS/HA/Si-HPMC hydrogels and the CS/HA/Si-HPMC hydrogels at different concentrations (50 μg/mL at 300 µg/mL) with the plate were further incubated for 24 h under the same conditions. The supernatant was extracted and dimethyl sulfoxide (DMSO) was homogenized with the samples using a glass tissue grinder (WHEATON, USA) to dissolve the crystals of formazan. The cells were incubated again for 4 h, and with 100 μL of DMSO, the formation of formazan violet was resolved. A serum-free medium acted as a monitor in this research. On a multi-well plate reader (Thermo Science, FC Multiskan), the absorbance was read at 570 nm. As positive and negative cytotoxicity monitors, respectively, DMSO and supplemented culture medium were used. Briefly, the 10^5^ cells were added into a 96 well plate directly to 100 μL of each hydrogel formulation. The kit was grown for 24 and 48 h in a 100 μL supplemented DMEM medium. The material was then washed in each well with PBS. After 24 and 48 h in culture, all of the medium in the wells was removed. The samples were then observed and imaged with an optical microscope.

### *In vivo* and histological staining study

All animals were treated under the Medical Animal Care Guidelines of the Medical College of Shanghai Jiaotong University. Under anesthesia (xylazine:ketamine = 5:1, v/v), each hydrogel (0.1 mL) loaded with 1 μg hydrogels were subcutaneously injected into the dorsal side of the mice using a 29-gauge needle. The surgery has proceeded under general anesthesia, and all attempts were made to minimize animal suffering. To evaluate the biocompatibility, control hydrogels without cells were transplanted into the back subcutaneous tissue of mice anesthetized (*n* = 3) by isoflurane inhalation. To confirm the safety of the fabricated hydrogels with a 3 mm height and 6 mm diameter size was implanted into the subcutaneous parts of the Sprague–Dawley rat. The CS/HA/Si-HPMC composite hydrogel with 4 × 8 mm^2^ in size was implanted into the muscle pouch. After 2 and 3 weeks of implantation, three rats were sacrificed every time, and the hydrogel samples were harvested. The specimens were frozen with CryoMatrix in liquid nitrogen. The three specimens were fixed with 4% formaldehyde for 4 days, then dehydrated in 50%, 75%, and 100% ethanol and embedded with paraffin. The specimens were cut into slices of 5 μm in thickness and stained with H&E and Masson’s trichrome for observation by light microscopy (Olympus, Japan).

*In vivo* study in a cartilage defect was performed with a Sprague–Dawley rat (6-weeks-old, female) were randomly divided into two groups: CS/HA and CS/HA/Si-HPMC (*n* = 3 for each group). The cartilage was exposed (diameter of 6.5 mm, 4 mm deep) injury was created by using a 1 mm drill. Encapsulation of cells in the hydrogels method and injected in the defect area. 3 weeks after implantation, the implanted hydrogels were removed, and then fixed in 4% PFA, embedded in paraffin, and sectioned transversely into 14 μm-thickness sections. The prepared decalcified tissues were dehydrated by treating a series of ethanol molded in a paraffin block. After incubation for different periods up to 3 weeks, the cell-seeded gels were washed at room temperature with PBS and fixed for two days with 10% neutral buffered formalin. The chondrocyte-gel constructs were cultured in a chondrogenic medium for 3 weeks for *in vitro* cartilage regeneration and subcutaneously implanted into nude mice for further 5 or 7 weeks. All *in vitro* and *in vivo* samples (*n* = 3) were harvested for cartilage regeneration evaluation. The sections were stained with Safranin-O for cartilage matrix observation. Histology analysis was carried out using the areas positively stained by Safranin-O in the defects and was scored. Masson’s trichrome and hematoxylin/eosin (HE) staining were also performed for reference. The parts were eventually visualized (Zeiss Axioplan 2, Göttingen, Germany) using an optical microscope. All reagents were purchased from Sigma-Aldrich except for hematoxylin which was obtained from Fisher Scientific, Hampton, USA.

### Statistical analysis

The mean ± standard deviation (SD) of all data was presented. By unidirectional analysis of variance (ANOVA), statistical variation was analyzed. A difference between the mean values of each category, if the *p*-value was less than .05, was considered statistically significant.

## Results and discussion

### Hydrogel morphology

**Figure 1.**
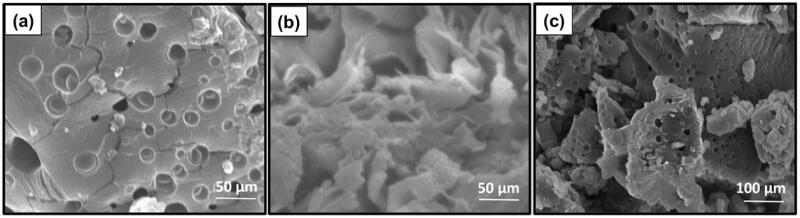
Morphological analysis (FE-SEM) of the (a) CS/HA, (b) Si-HPMC, and (c) CS/HA/Si-HPMC hydrogels.

To obtain the information on the microscopic structure, the hydrogels were lyophilized for morphological observation by scanning electron microscopy (SEM). The scaffolds were highly porous with a network of interconnected pores produced by freezing and lyophilization in the solvent (Engkagul et al., 2018), as shown in Figure 1. Compared with CS/HA hydrogel and Si-HPMC, the CS/HA/Si-HPMC composite hydrogels have a more condensed structure and the pore size decreases with the Si-HPMC (Figure 1(a–c)). Furthermore, no clear biphasic structure can be observed when Si-HPMC is involved, indicating that Si-HPMC might interfere with the excellent interaction of CS and HA molecular chains. These findings suggested that there were ample pathways for the CS/HA/Si-HPMC hydrogel to ensure cell attachment and migration (Iqbal et al., 2017).

### FTIR analysis

**Figure 2.**
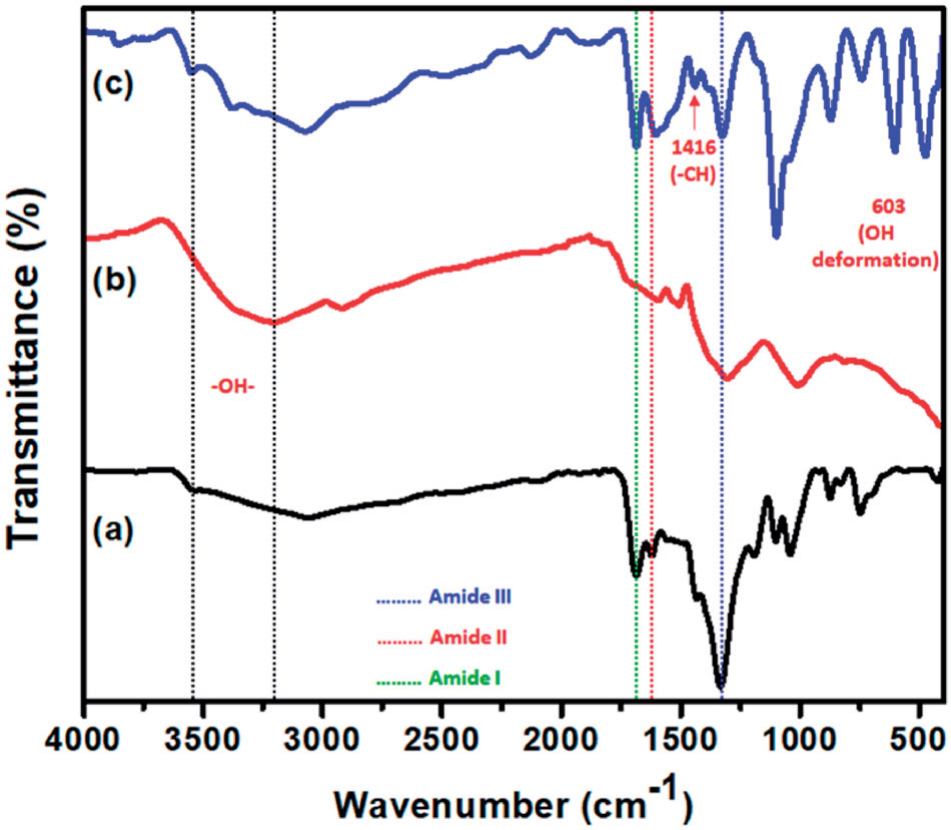
FTIR spectra of the (a) CS/HA, (b) Si-HPMC, and (c) CS/HA/Si-HPMC hydrogels.

To determine the chemical structure of hydrogels, FTIR spectroscopy was performed and the potential interaction between CS and HA (CS/HA) and the composite hydrogel CS/HA/Si-HPMC is shown in Figure 2. The CS/HA composite revealed the CS peaks at 1591 cm^−1^ (bending –NH_2_), 1379 (amide III), and 1636 cm^−1^ (amide), indicating the presence of HA in the composite hydrogel. The slight shift in amide bonds can be attributed to interactions between polysaccharides, confirming complexation. Hydroxyl groups (O–H) and hydrogen bonds occurring in uncrosslinked HA and CS/HA hydrogel, respectively, are the large bands that appear at 3275 cm^−1^ and 3269 cm^−1^ (Bazmandeh et al., 2019). The lack of a peak indicated the lack of an unreacted carbonyl group in the 1724 cm^−1^. The interaction between CS and HA through the CS amide group and the free HA hydroxyl groups is indicated by this change (Tan et al., 2009). For the O–H and N–H stretch vibration peaks, the development of the Si-HPMC and CS/HA hydrogel crosslink has also resulted in reduced absorbance.

Furthermore, the absence of a peak in the 1620–1750 cm^−1^ suggested the absence of an unreacted carbonyl group. The vibration of hydroxyl ions in Si-HPMC reflects the peak observed at 603 cm^−1^. Besides, two other bands were identified in each spectrum, 1416 cm^−1^ and 1319 cm^−1^ for Si-HPMC, which coincide with the symmetrical stretching of the carboxylate of HA and CS C–N, respectively. Due to the ionic interaction between the negatively charged HA and the positively charged CS amino group, the lack of the characteristic absorbance peak at 1172 cm^−1^ suggested that the CS/HA hydrogel complex had occurred. All the characteristic peaks moved between Si-HPMC and the CS/HA hydrogel composites in the IR spectra of Si-HPMC suggested that the chemical reaction took place (Jeong et al., 2016). The results suggest that spontaneous interactions between the CS/HA hydrogel and Si-HPMC generated an ionically cross-linked network structure.

### XRD analysis

**Figure 3.**
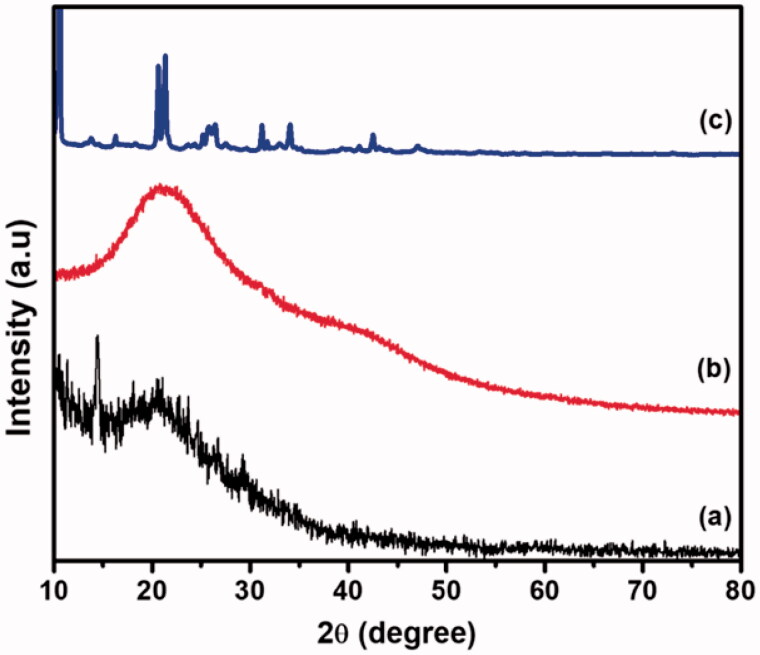
XRD pattern of the (a) CS/HA, (b) Si-HPMC, and (c) CS/HA/Si-HPMC hydrogels.

The XRD diagram of the CS/HA, Si-HPMC, and CS/HA/Si-HPMC hydrogel composition is shown in Figure 3. The XRD diagram of pure CS gives two characteristic peaks at 2*θ* = 10.42° and 2*θ* = 19.99°, respectively (Kumar & Koh, 2012) In the case of HA, two peaks at 10.23° and 20.45° according to the previous literature (Lopez et al., 2020). The characteristic peaks were formed at approximately 2 = 15.34° and 21.71°, showing that the addition of HA modified the CS structure due to the formation of stronger hydrogen bonds between amino groups and hydroxyl groups (Zhang et al., 2017). The crystallinity of the CS/HA/Si-HPMC hydrogel, determined by XRD diffractometer, showed that the peaks at 2*θ* = 10.52° indicate the presence of Si-HPMC, and the peaks at 2*θ* = 24.24°, 29.98°, 32.27°, 35.64°, and 42.21° mainly attributed to strong molecular interaction (Liu et al., 2014). The presence of additional peaks in the small angular region can be attributed to the formation of Si-HPMC with an interpenetrating polymer lattice structure that, due to a more complex structure, causes a shift in crystallinity. This evidence indicated that the reaction had taken place between the Si-HPMC hydrogel and CS/HA in the mixing solution and thus significantly strong interactions were formed, altering the crystal microstructure of the CS/HA/Si-HPMC hydrogels. It was consistent with the FTIR findings.

### TGA analysis

**Figure 4.**
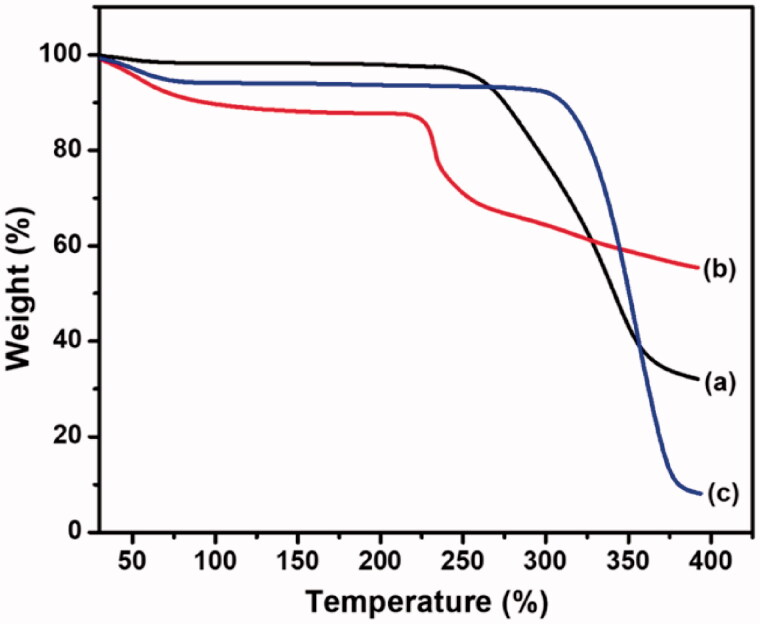
TGA analysis of the (a) CS/HA, (b) Si-HPMC, and (c) CS/HA/Si-HPMC hydrogels.

TGA measurements for the CS/HA, Si-HPMC, and CS/HA/Si-HPMC hydrogels were revealed in Figure 4. The first phase of decomposition has been attributed to the loss of water and moisture content of the CS/HA hydrogels, which were released in the range of about 50.0–110.0 °C. This phenomenon has been attributed to the degradation of HA and has been implicated in the partial disruption of molecular structure. Due to the structure of CS, the water molecule can be linked by two polar groups, namely when hydroxyl and amine are added due to cross-linked or HA (CS/HA) (Zhang et al., 2017; Bazmandeh et al., 2019). The second decomposition step of the CS/HA/Si-HPMC hydrogels revealed a weight loss of about 350.0–400.0 °C for the hydrogels with Si-HPMC, respectively. It should be noted that due to the increase in Si-HPMC (240.0 °C), the maximum weight loss stages of the hydrogels tended to shift the peak maximum to higher temperatures, reflecting the improvement of thermal CS/HA/Si-HPMC hydrogels stability compared to CS/HA hydrogels without Si-HPMC (Rotta et al., 2011; Chuysinuan et al., 2020). The reason for this improvement in thermal stability is the strong interaction of Si-HPMC with the CS/HA hydrogel polymer matrix (CS/HA/Si-HPMC) which reduces the mobility of the side chains of the polymer.

### Mechanical, rheological, and compression properties of hydrogels

**Figure 5.**
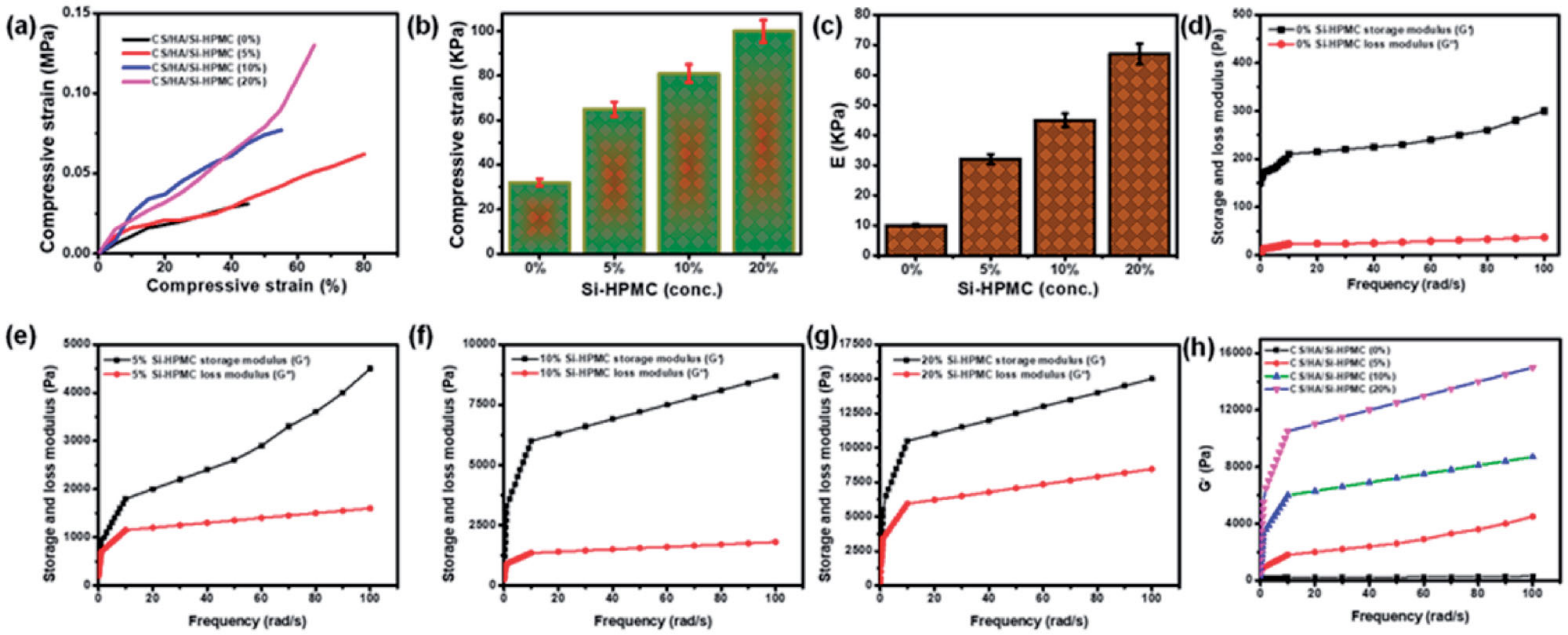
(a) The representative compressive strain-stress curves of different hydrogels. (b) The compressive strength of different hydrogels. (c) The compressive elastic modulus of different hydrogels. (d–g) Storage modulus (G′) and loss modulus (G′′) of hydrogels without Si-HPMC hydrogels treatment and Si-HPMC hydrogels-treated hydrogels (0%, 5%, 10%, and 20%). (h) Storage modulus (G′) of different hydrogels. **p < .01.

To demonstrate the feasibility of the use in the application of cartilage, to better understand the effect of mixing CS with HA and also the use of Si-HPMC on the mechanical properties of hydrogels were carried out. The representative compressive stress-strain curves have been shown in Figure 5(a). It has been noted that the introduction of Si-HPMC into CS/HA hydrogels greatly improves the mechanical properties of CS/HA/Si-HPMC. Specifically, when compared to CS/HA hydrogels, Si-HPMC reinforced CS/HA/Si-HPMC hydrogels showed remarkable improvement in compressive strength and elastic modulus in compression due to crosslinking density higher (Figure 5(b,c)) (Tan et al., 2009). As shown in Figure 5(b), the compressive strength increased from 30 ± 6 kPa (CS/HA) to 99 ± 4 kPa (CS/HA/Si-HPMC), with an increase of approximately 3.5 times. The key explanation is that the structure of this hydrogel uses no crosslinking agent. Another explanation is that HA is integrated into the hydrogel structure. Since HA is very hydrophilic and decreases the shrinkage of the hydrogel, the compressive module gel has a lower value (Liu et al., 2020; Wang et al., 2020). Regarding the elastic compressive modulus, a similar trend was observed with Si-HPMC in hydrogels (Figure 5(c)), showing a 7-fold increase. However, the stiffness of the hydrogel matrix can be modulated by changing the concentration of the Si-HPMC component in the hydrogel.

To determine whether hydrogels could be used in clinical applications, the viscoelastic properties of CS/HA/Si-HPMC systems were evaluated with different Si-HPMC systems by measuring the storage modulus (G′), which is the behavior of the material and modulus of loss (G′′), indicative of viscous behavior. All groups showed that G′ was much higher than G′ without a trace of interception when the oscillating shear stress was set at 5% and the angular frequency increased from 0.1 to 100 rad/s (Figure 5(d–g)), indicating the predominantly elastic nature of the hydrogels produced. The fracture energy and the compressive modulus of CS/HA and CS/HA/Si-HPMC hydrogel composites (Nguyen et al., 2019). As prepared, the CS/HA/Si-HPMC hydrogel has greater compressive strength than the containing CS/HA hydrogel (Gilarska et al., 2018). This is probably attributed to the fact that the Si-HPMC introduced prevented the formation of CS/HA and, therefore, reduced the structural density of the hydrogels. Besides, the CS/HA/Si-HPMC hydrogels have double cross-linked structures and are rich in bound water, which has clear implications for the compressibility of the hydrogels and the propagation of the concentrated CS/HA stress region. More importantly, Figure 5(h) showed that the G′ of the CS/HA/Si-HPMC hydrogels enhanced with Si-HPMC gradually increased with increasing Si-HPMC concentration and was significantly higher than that of the hydrogels without Si-HPMC with the increased Si -HPMC concentration implying the existence of more stable and rigid lattice systems in the CS/HA/Si-HPMC hydrogels reinforced with Si-HPMC. This meant that Si-HPMC could help increase hydrogel strength and also provided direct evidence for the interaction of Si-HPMC with CS/HA hydrogels. This indicates that Si-HPMC can cause a reaction very quickly. Thus, the comparison of the modulus of the characteristic elasticity values of all the hydrogels of the rheological experiment can serve as proof that the materials prepared can be used as injectable hydrogels (Ghorbani et al., 2020). Overall, the combination of mechanical, rheological, and compressive properties has proven to promote Si-HPMC on mechanical performance and Si-HPMC reinforced CS/HA hydrogels would be more suitable for cartilage application.

### Swelling ratio and in vitro degradation

**Figure 6.**
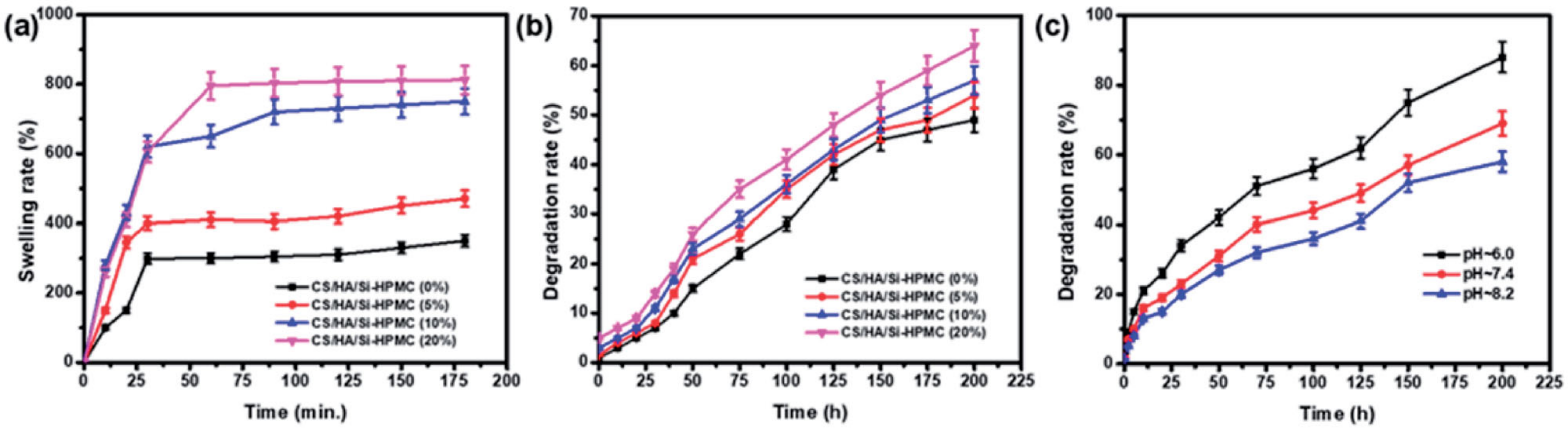
The swelling ratio of different hydrogels of hydrogels at pH = 7.4 (a), the degradation rate of different hydrogels at pH = 7.4 (b), the degradation rate of the CS/HA/Si-HPMC at pH∼6.0, 7.4 and 8.2, respectively (c).

The structures of the CS and HA copolymers, the hydrogels synthesized have a high-water retention capacity. There are amine and hydroxyl groups in the structure of CS and HA, which have a great ability to create hydrogen bonds. Amino groups are expected to be efficiently incorporated into the polymer network, which can increase network density in hybrid materials. The swelling ratio of the CS/HA/Si-HPMC hydrogel composite is shown in Figure 6(a). The impact of Si-HPMC differs depending on the CS/HA weight ratio. Subsequently, the swelling rate decreased with the rise in Si-HPMC concentration, which is due to the decrease of CS/HA in the CS/HA/Si-HPMC hydrogel, and the cross-linked Si-HPMC made the network denser (Thomas et al., 2017; Chuysinuan et al., 2020). The ability of the material to retain water is highly dependent on both hydrophilicity and microstructure. In conclusion, the results of swelling tests showed that increasing the concentration of Si-HPMC in CS/HA hydrogels improves the efficiency and stability of crosslinking, which can make this type of hydrogel more stable *in vitro* and *in vivo* in an aqueous medium.

To examine the degradation behavior of hydrogels in a physiological state, an *in vitro* degradation test was performed and the results were shown in Figure 6(b). During the experiment, the degradation of the composite hydrogels was followed as a function of the incubation time in PBS medium at 37 °C and of the interaction with the surface of the hydrogel. The degradation behavior of hydrogels is also related to the mass ratio of CS/HA and Si-HPMC (Park et al., 2013). To further determine the hydrogel degradation associated with the breaking of the C=N bond, the degradation ratio of the CS/HA/Si-HPMC hydrogel was studied at different pH values. As shown in Figure 6(c), the hydrogel shows the controlled and sustained rate of degradation to PBS with a pH of 6.0. It indicates that the cleavage of the C=N bond in an acidic environment promotes the degradation of the CS/HA/Si-HPMC hydrogel (Liu et al., 2020). The degradation rate of the hydrogel in the decreasing pH (6.0) indicated that CS/HA/Si-HPMC provides increasing degradation ability. In addition, Si-HPMC enhanced CS/HA hydrogels provided a controlled and sustained degradability rate with increasing Si-HPMC ratio in the hydrogel, which could extend the functional life of tissue engineering applications.

### Cytotoxicity test

**Figure 7.**
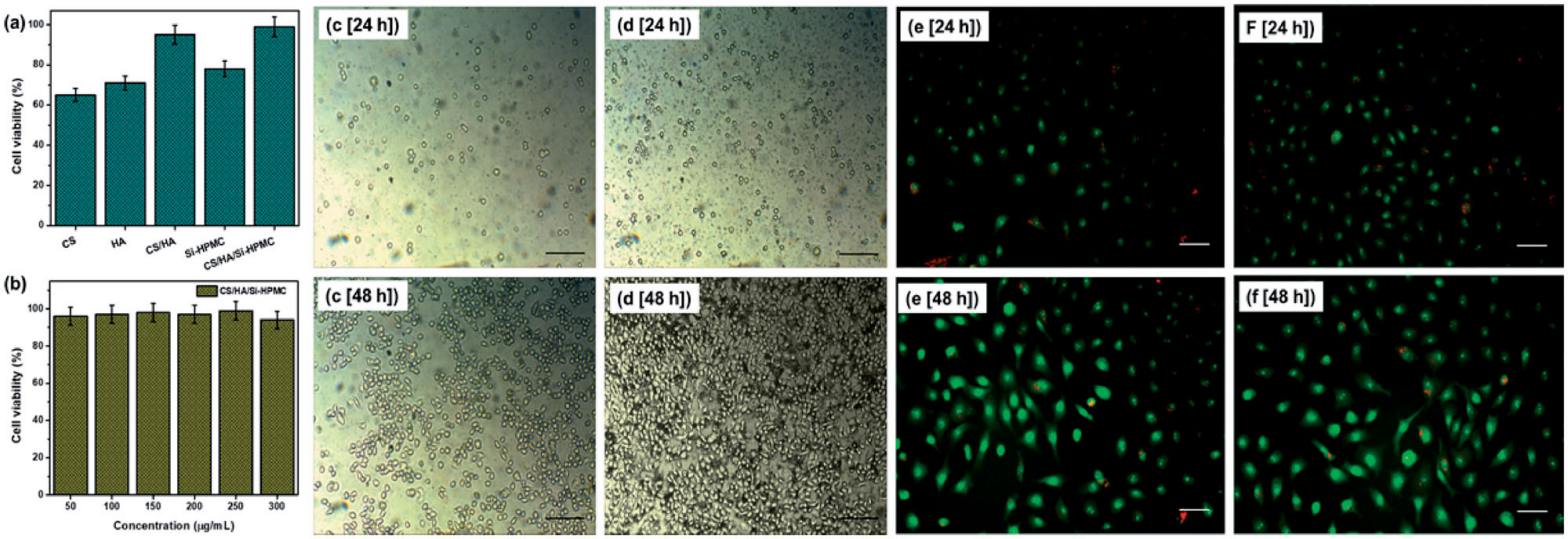
The cell viability of different hydrogels at a concentration of 250 μg/mL (a), the cell viability of the hydrogel at different concentrations (50–300 μg/mL) (b), L929 cells multiply on different CS/HA hydrogel surfaces (c, e) and CS/HA/Si-HPMC hydrogel surfaces (d, f) at different times (24 h and 48 h). (Scale bar = 100 μm).

The MTT tests are carried out on prepared CS, HA, CS/HA, and Si-HPMC, CS/HA/Si-HPMC hydrogel composites. Figure 7(a,b) shows the proliferation and viability of cells by the MTT test. The viability of L929 cells incubated for 48 h in a medium with the same content of pure matter and CS/HA and CS/HA/Si-HPMC hydrogels was between 65% and 95%, indicating that the good compatibility of the CS/HA/Si-HPMC hydrogels. However, the CS/HA and CS/HA/Si-HPMC hydrogel formulations had cell viability greater than 90% for both hydrogel extract concentrations, indicating that these hydrogels were not cytotoxic and that differentiation and cell growth. Therefore, the CS/HA/Si-HPMC hydrogel products made in various contents of this study are biocompatible and can be used as excellent platforms for cell growth *in vitro* (Viguier et al., 2016; Zhu, Tan, et al., 2017; Vignesh et al., 2018).

Based on these results, none of the CS/HA/Si-HPMC hydrogels harmed cell growth and should be tissue safe. Biocompatibility is known to be the most essential performance for CS/HA and CS/HA/Si-HPMC hydrogels used as excipients. Thus, it is necessary to assess the growth of cells in the two-dimensional spaces of hydrogel carriers. Figure 7(c,d) shows the proliferation of L929 cells on the surface of CS/HA and CS/HA/Si-HPMC hydrogels for 24 h and 48 h. Significant differences are found between the CS/HA and CS/HA/Si-HPMC samples after 48 h of cell culture. Cellular viability demonstrates the biocompatibility of CS/HA/Si-HPMC with L929 cells, making it a potential candidate for tissue engineering applications.

### *In vitro* and *in vivo* studies for cartilage regeneration

**Figure 8.**
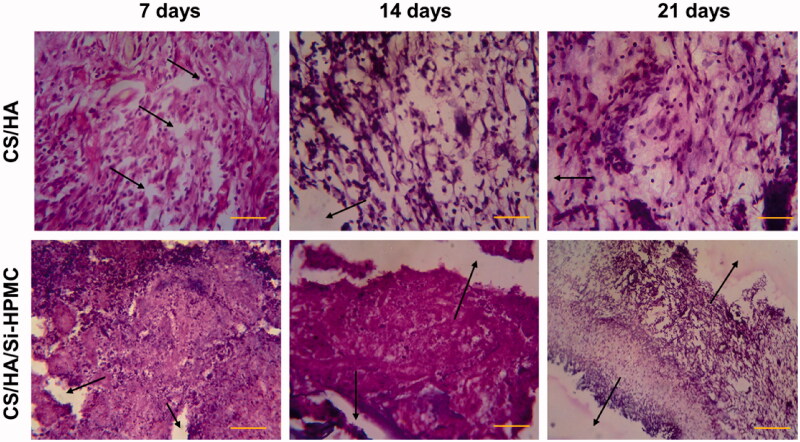
Histological sections of subcutaneous mouse tissue after implantation of CS/HA and CS/HA/Si-HPMC hydrogels for 7, 14, and 21 days and the strong integration (represented by arrows). (Scale bar = 100 μm).

**Figure 9.**
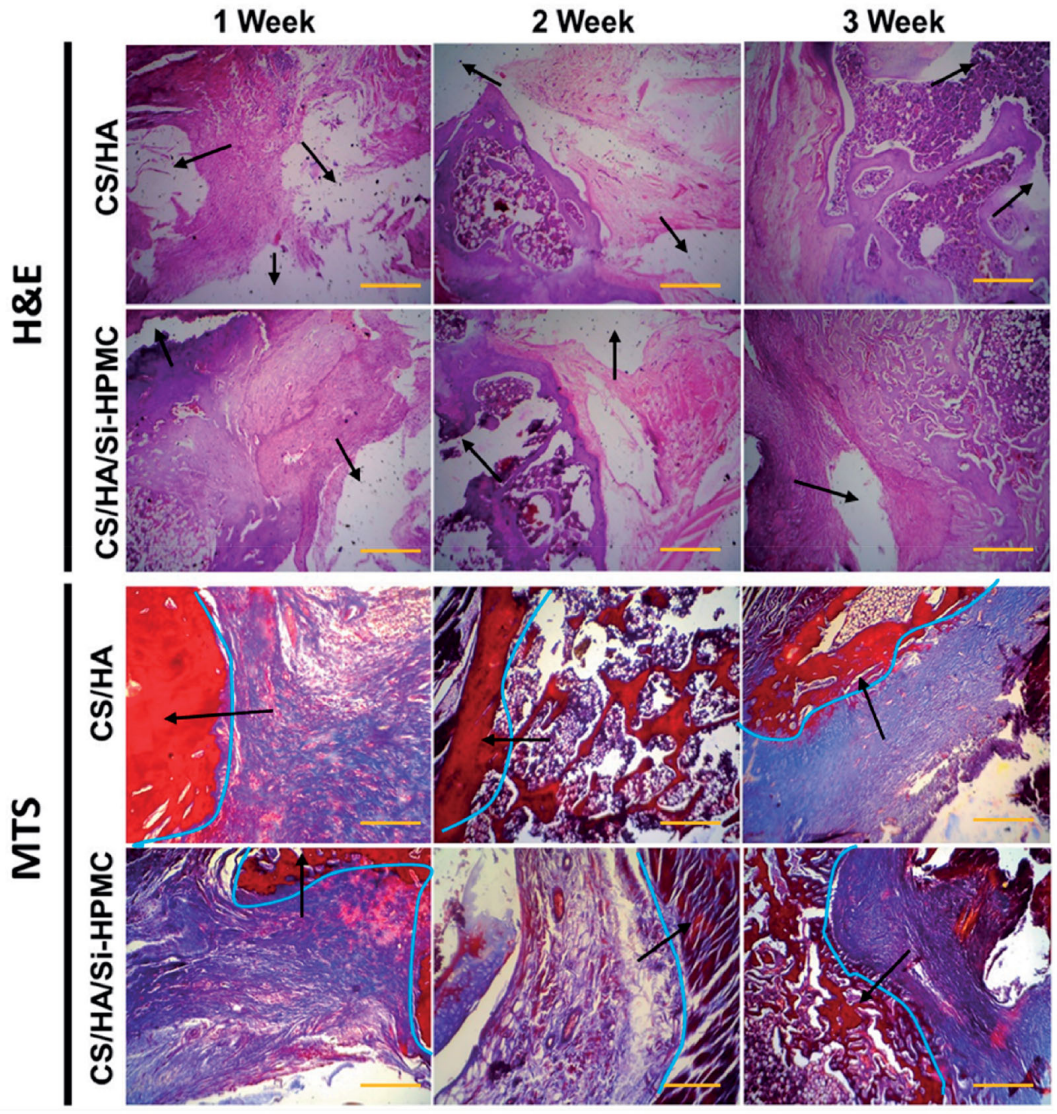
Histological sections (H&E staining and MTS staining) of cartilage formed by chondrocytes encapsulated with CS/HA hydrogels and CS/HA/Si-HPMC hydrogels and the strong integration (represented marks by arrows and line curves). (Scale bar = 100 μm).

**Figure 10.**
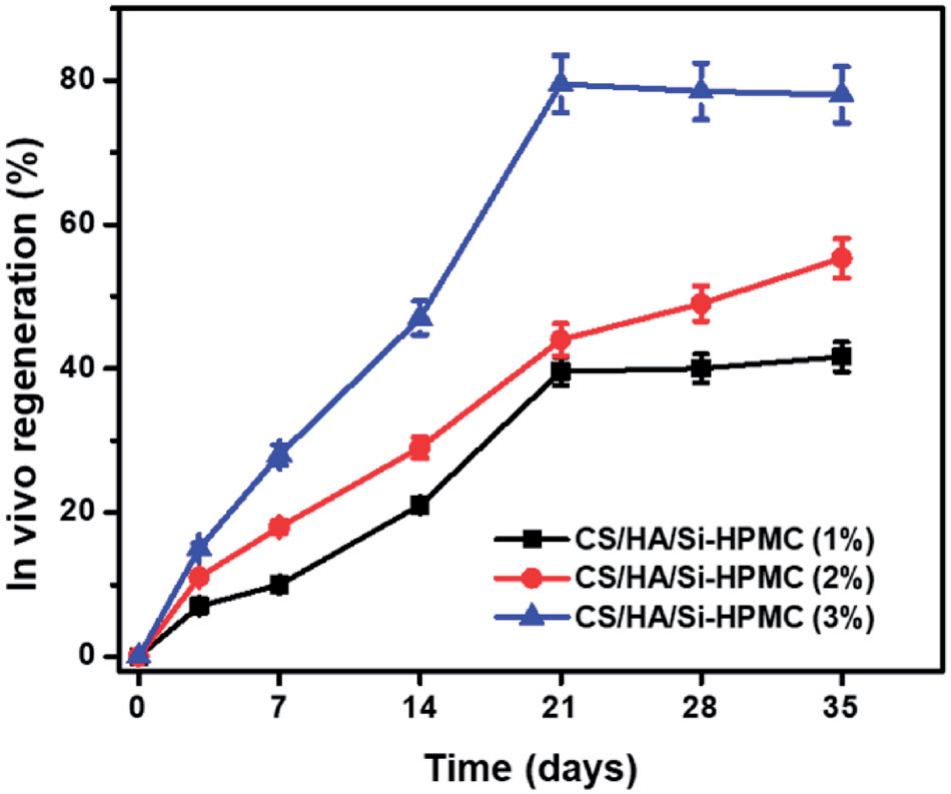
*In vivo* bone regeneration of the CS/HA/Si-HPMC hydrogels in different time periods. Data are presented as the mean ± SD (*n* = 3).

The above results suggest that hydrogels with controllable mechanical, rheological, and compressive properties, rate of swelling, rate of degradation, and cellular cytotoxicity could potentially be useful as an injectable scaffold for various tissue regeneration applications. More importantly, the inflammatory response elicited by the CS/HA and CS/HA/Si-HPMC hydrogels was studied histologically by hematoxylin-eosin (H&E) staining, with the inflammatory cell nuclei stained purple and normal on fabric is pink in color (Deng et al., 2017). Representative images of hematoxylin and eosin and trichrome staining of regenerated cartilage with an average or low score in animals receiving CS/HA and CS/HA/Si-HPMC hydrogels as implant are illustrated in Figures 8–10, respectively. On day 7 after implantation, the inflammation of the CS/HA/Si-HPMC hydrogels was slightly worse than that of the CS/HA hydrogels. However, on day 7, the CS/HA/Si-HPMC hydrogels significantly reduced the inflammatory response, which was almost resolved by day 14. Also, the CS/HA/Si-HPMC hydrogels on day 21 had better resistance than the CS/HA/Si-HPMC hydrogels from day 14 to the inflammatory response (Zhang et al., 2014; Deng et al., 2017; Vignesh et al., 2018). It is worth noting that the number of nodules increases when more CS/HA is added during the mixing process. These results indicate that Si-HPMC with CS/HA supports the formation of cartilaginous tissue in rats when implanted with cells. This result indicated that increasing the concentration of Si-HPMC was very effective in preventing an inflammatory response.

In this study, the hydrogel with CS/HA with Si-HPMC hydrogels was used for the regeneration of cartilage. Each histological result showed that cartilage cell structure and neo-cartilage formation appeared in CS/HA/Si-HPMC hydrogels with chondrocytes of human origin from one-week-old cultures. In H&E staining and MTS, the number of chondrocytes increased over periods of the culture of 1 to 3 weeks, and the population of chondrocytes gradually increased according to the period of culture. Cartilage in CS/HA/Si-HPMC hydrogels showed that more chondrocytes formed cartilage in this hydrogel than in CS/HA hydrogels (Figure 9) (Vinatier et al., 2005). In contrast, there were signs of inflammation around the newly formed cartilage tissue in the CS/HA hydrogels (Kim et al., 2019). Chondrocytes were observed in the pores on pink-colored CS/HA/Si-HPMC hydrogels, and the surrounding matrix was gradually stained depending on the period of culture at the time of staining. The incorporation of Si-HPMC can help to form a more homogeneous cement matrix, which may contribute to the improved fracture toughness. However, compared to these fiber-containing cement, Si-HPMC composite CS/HA hydrogels are easier to process regarding injectability, cohesion, and polymer distribution, and therefore appear to be an attractive alternative. In particular, the implanted group of CS/HA/Si-HPMC hydrogels showed densely regenerated tissue and interacted well with the surrounding tissue (Zhang et al., 2016). In particular, the strong integration (represented by arrows) between the CS/HA/Si-HPMC hydrogels and the surrounding tissues was observed, confirming that the existence of Si-HPMC can improve the regeneration of cartilage (Li et al., 2006; Liuyun et al., 2009).

The *in vivo* bone regeneration ratio of CS/HA hydrogels with Si-HPMC (1, 2, and 3%) in PBS has been investigated in the present study and the findings are shown in Figure 10. The regeneration curves of CS/HA/Si-HPMC, indicating their relatively steady regeneration, are similar to a straight path with long retention periods. For instance, at 14 days and 21 days, CS/HA/Si-HPMC(3%) could be regenerate as early as 7 days, and their regenerate rates produced nearly 41.6% and 55.3%, respectively. The regeneration rate of the CS/HA/Si-HPMC(3%) hydrogel reached about 79.5% at 21 days for long retention periods, indicating relatively good *in vivo* bone regeneration. After 21 to 35 days of regeneration, bone regeneration rates for all hydrogels were above 50%. In this respect, once bone healing is complete, the prepared hydrogels can provide preserved mechanical stability and can then be progressively replaced spontaneously by new regenerative bone tissue. Although this study showed that the amount of bone regeneration of control sites was higher than nanocomposites hydrogels, the CS/HA/Si-HPMC hydrogel also showed reasonable bone in-growth and can be a good candidate to replace natural bone regeneration and to maintain space for a longer period of time. Our studies suggest that the combination of growth factors has been reported to induce rapid and successful bone-tissue regeneration. These CS/HA/Si-HPMC hydrogels are promising candidates for tissue compatibility injectable scaffolds. Based on these results, CS/HA/Si-HPMC hydrogels can provide an excellent environment for the growth and maintenance of chondrocytes. The data provide proof of the principle that the resulting hydrogel has an excellent ability to repair joint cartilage using a tissue-engineered approach. However, we could not rule out the integration between the newly formed cartilages, which merits further investigation. To understand the initial response and long-term performance of the hydrogel for cartilage regeneration, to understand in-depth the degradation of the hydrogel *in vivo*, and to provide quantitative biochemical estimates and thorough functional analysis of the regenerated tissues, we propose future studies in small and large animals.

**Figure 11.**
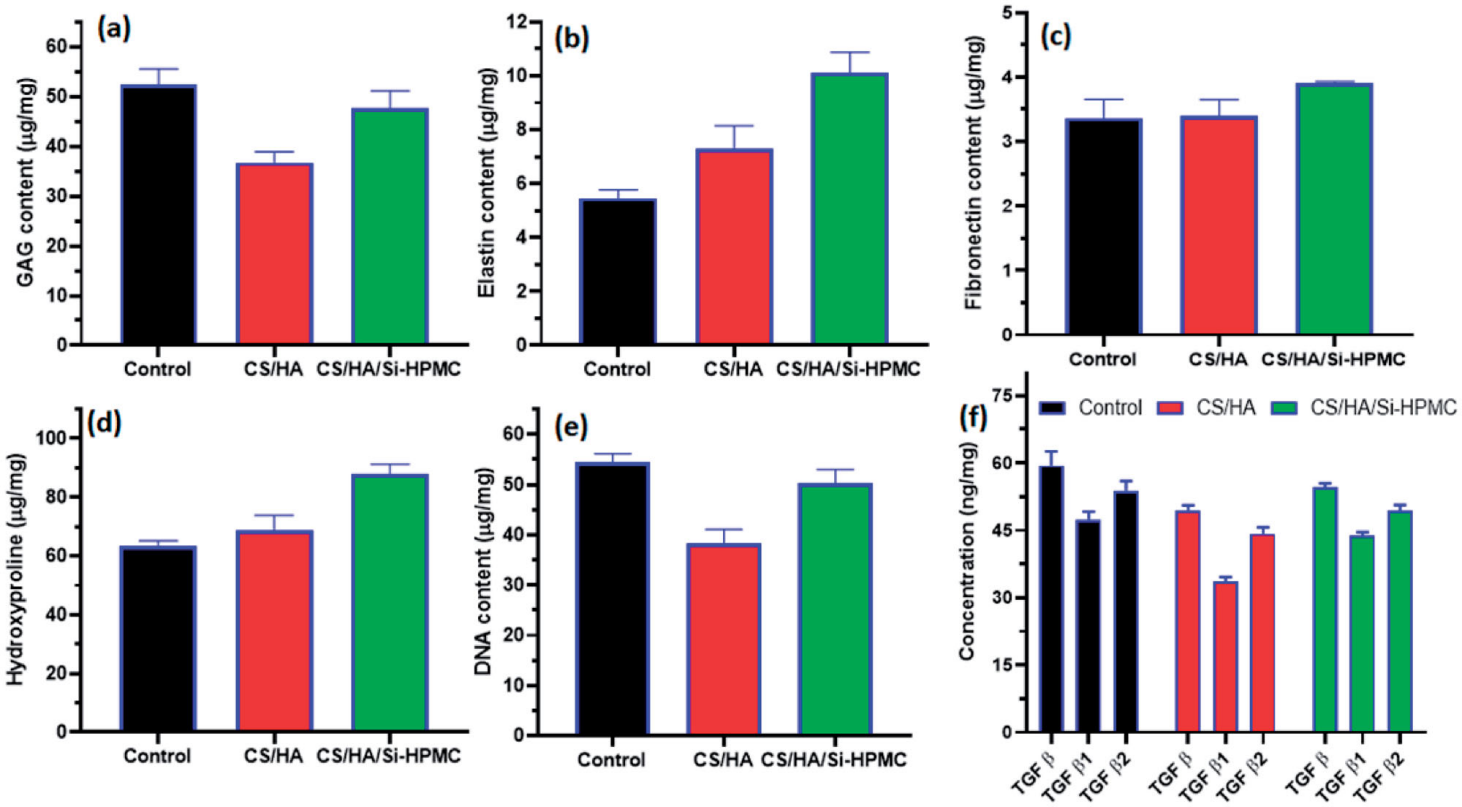
Quantitative analysis of biochemical assay on cartilage implanted *in vivo* samples of CS/HA, CS/HA/Si-HPMC and control (without any implant) exhibited the results of contents of GAG (a), Elastin (b), fibronectin (c), collagen (d), DNA (e) and TGF β, β1, & β2 (f). Data value described as mean ± SD (*n* = 3).

*In vivo* quantitative biochemical evaluations of treated CS/HA and CS/HA/Si-HPMC samples were done after treatment of 35 days by using specific GAG, DNA, collagen, fibronectin, elastin, and TGF β, β1, β2 kit experimental protocols (Figure 11). After 35 days of implantations, treated tissue groups (CS/HA and CS/HA/Si-HPMC) were expunged out for the biochemical studies. The healthy tissue group (no implant and treatment) was used as a control group. The obtained quantitative results of GAG, DNA, collagen, fibronectin, and elastin groups analysis established that outcome of CS/HA/Si-HPMC samples have more similar values to control groups and also much improved results when compared to CS/HA samples treated group, which reveals better host-graft orientations between treated groups and nature cartilage tissues. Particularly, the content of GAG in CS/HA/Si-HPMC has exhibited a very similar value and statistically insignificant with control groups (Figure 11(a)). Nevertheless, the observation of collagen content (Figure 11(d)) was suggestively increased in the CS/HA and CS/HA/Si-HPMC treated groups when compared to the control (without any implant), which was correspondingly exhibited by the histological (MTS) observations. These observations have proved the healthy organization and matured collagen fibers formations after treatment. Likewise, the quantitative data of fibronectin (Figure 11(c)) and elastin (Figure 11(b)) significantly enhanced in the CS/HA/Si-HPMC sample treatment when compared to the CS/HA and control groups, which demonstrated improved materials–cell interactions after implantations of prepared host materials. The values of TGFβ, β1, and β2 were estimated through ELISA kit showed comparable ranging results and no very significant difference from the control groups as shown in Figure 11(f) (Asadi et al., 2019; Chen et al., 2020; Yu et al., 2020).

## Conclusions

In this study, a novel water-soluble CS/HA with silanized-hydroxypropyl methylcellulose (Si-HPMC) injectable hydrogel composite was designed for tissue engineering of cartilage. Si-HPMC was homogeneously distributed into CS/HA hydrogel system and had beneficial effects on improving mechanical properties, stabilizing the network of hydrogels, and slowing down weight loss at the expense of reducing gelation time and swelling ratio. The amount of Si-HPMC(3.0%) in the CS/HA hydrogel affected the surface morphology and the pore size of the composite scaffolds. The physicochemical properties of the hydrogel network morphology, surface properties, swelling rate, degradation behavior, and mechanical, rheological, and compressive properties can be manipulated by varying the weight ratio and concentration of the CS/HA hydrogel and Si-HPMC. Besides, L929 cells were viable and proliferated well on the CS/HA/Si-HPMC hydrogel during the period of *in vitro* cell culture. The existence of HA, one of the key components of cartilage, maybe due to this biocompatibility. *In vitro* chondrocytes seeded in the composite CS/HA/Si-HPMC hydrogels actually show significantly higher viability during culture time up to 21 days. These results demonstrated that these CS/HA/Si-HPMC hydrogels, especially the one consisting of 3% (w/v) Si-HPMC, exhibited the best comprehensive properties for bone regeneration applications. The use of CS/HA/Si-HPMC hydrogels in subcutaneous or nude mice in conjunction with chondrocytes has shown their possible use for cartilage tissue engineering. Therefore, we believe that this injectable CS/HA hydrogel loaded with Si-HPMC is a promising tissue engineering strategy and has great potential for the clinical repair of cartilage tissue.
